# Takayasu's Arteritis and Its Association With Mycobacterium Tuberculosis: A Systematic Review

**DOI:** 10.7759/cureus.16927

**Published:** 2021-08-05

**Authors:** Manusha Thapa Magar, Sunam Kafle, Arisa Poudel, Priyanka Patel, Ivan Cancarevic

**Affiliations:** 1 Internal Medicine, California Institute of Behavioral Neurosciences & Psychology, Fairfield, USA; 2 Internal Medicine/Neurology, California Institute of Behavioral Neurosciences & Psychology, Fairfield, USA

**Keywords:** takayasu's arteritis, mycobacterium tuberculosis, tuberculosis, systematic review, granulomatous vasculitis

## Abstract

Takayasu's arteritis (TAK) is a rare large vessel vasculitis of unknown etiology that chiefly targets the aorta and its branches. It predominantly affects females under 50 years of age. A relationship between TAK and* Mycobacterium tuberculosis* (TB) has been suggested for a long time, but only a few systematic studies have been done centering on this association. The present systematic review aimed to analyze the possible association between TAK and TB based on the studies conducted previously. A detailed search was conducted until April 2021 using three databases: PubMed, Cochrane Library, and MedlinePlus. PubMed search on the related topic identified 1053 articles, four on Cochrane Library, and three on MedlinePlus. Finally, 13 papers were pertinent for our review. The appropriate data was extracted from these articles, and the risk of bias assessment was done. The systematic review of these finalized articles found that the majority of the current studies supported the presence of TB in patients with TAK. Out of 13 final observational studies, only one study failed to detect a link between TAK and TB. However, data are still lacking that show a direct link between them. Future large-scale studies are needed to probe the exact role of *Mycobacterium tuberculosis* infection in the etiopathogenesis of TAK.

## Introduction and background

Takayasu’s arteritis (TAK), also known as “pulseless disease," is an uncommon, chronic granulomatous vasculitis that mainly affects the large arteries such as the aorta and its primary branches [1]. It was initially described in 1908 by Dr. Mikito Takayasu, a professor of ophthalmology at Kanazawa University, Japan [2]. One in 200,000 people is affected by TAK, predominantly affecting females under 40 years with a female to male ratio of 9:1 [1,2]. TAK occurs in every part of the world; however, it is more common in Southeast Asia, India, Japan, China, Korea, Mexico, and Latin America [1,3].

TAK can be present in two phases, a systematic phase followed by an occlusive phase [2]. The first phase shows non-specific constitutional symptoms such as fever, myalgia, fatigue, anorexia, weight loss, tenderness in the affected arteries [2,3]. The acute phase reactant such as erythrocyte sedimentation and C-reactive protein is usually raised in this phase [2,4]. The second phase occurs due to chronic inflammation and stenosis of the involved arteries, resulting in claudication of the limb, headache, dizziness, hypertension, chest pain, blood pressure discrepancies between two arms, and diminished or absent peripheral pulses [2,5]. It is characterized by the infiltration of inflammatory cells in tunica media, hyperplasia of the intima, and thickening of adventitia, histologically [6].

Tuberculosis (TB) is a curable and treatable disease that is distributed worldwide [7]. According to World Health Organization (WHO), in 2019, the most significant number of TB cases was seen in the WHO Southeast Asian region [7]. TB affects all age groups, adults being the most targeted population [7]. It is a transmissible bacterial infection caused by *Mycobacterium tuberculosis*, transmitted via the respiratory route that chiefly affects the lungs [8]. Nonetheless, other tissues and organs may also be involved [8]. Although one-fourth of the world's population is infected with tuberculosis, most of them only have latent tuberculosis within their lifetime; the rest of the affected individuals effectively contain their infection [8]. The risk of reactivation of latent to active tuberculosis is most significant in people with immune-deficient conditions [9].

The precise etiology of TAK continues to be unknown [1]. However, autoimmunity is mainly suggested as a cause of TAK [10]. The other causes that might contribute to TAK's etiopathogenesis are the genetic and infectious (bacterial, viral) causes [2,10]. Of the bacterial causes, the role of *Mycobacterium tuberculosis* has been implied [10]. TAK is one of the first vasculitides to be related to a particular infective organism [11]. Initially, this likely co-relation was mentioned due to the morphological resemblance of Langhans giant-cell granulomas with tuberculous lesions [12]. Another likely finding signifying this correlation is the evidence of tubercular lymph nodes in the arterial lesions, increased agalactosyl IgG level, augmented responses to purified protein derivative of *Mycobacterium tuberculosis* [10,13,14]. In addition, recent studies suggest the role of mycobacterial heat shock protein (HSP) in linking autoimmune disease and *Mycobacterium tuberculosis* [15]. The molecular cross-reactivity between host HSPs and mycobacterial HSPs could be the probable trigger for the autoimmune process [15]. Also, in TAK patients' aortic tissues, Soto et al. detected an increased frequency of IS6110 and HupB genes [16]. TAK is commonly seen in East Asia or Southeast Asia, where the prevalence of TB is high [17]. This systematic review aimed to examine and consolidate the relevant information on this connection. And, it intends to highlight the association between *Mycobacterium tuberculosis* and Takayasu’s arteritis and the possible cause for this link from the studies done previously. 

## Review

Methods

We conducted a systematic literature search following Preferred Reporting Items for Systematic Reviews and Meta-Analysis (PRISMA) guidelines [18]. The free full-text articles indexed in PubMed, Cochrane Library, and MedlinePlus were searched from April 10, 2021, to April 30, 2021, using the regular keywords "Takayasu's arteritis," "granulomatous vasculitis," "pulseless disease," "tuberculosis," "Mycobacteria tuberculosis," "TB," "active tuberculosis," "latent tuberculosis," "tubercul*” and medical subject headings (MeSH) terms "Takayasu Arteritis"[Majr] and "Tuberculosis"[Majr], alone and in combination. The detailed search strategy in three data sources is given in Tables 1, 2.

**Table 1:** Advance search strategy in PubMed MeSH: medical subject headings

S. No.	Combination of MeSH and Keywords	Search Results
1.	"Takayasu Arteritis"[Majr] OR “Takayasu’s arteritis” OR “Granulomatous Vasculitis” OR “Pulseless disease”	1053
2.	“Tuberculosis"[Majr] OR Tuberculosis OR “Mycobacteria Tuberculosis” OR “TB” OR “active tuberculosis” OR “latent tuberculosis” OR “tubercul*."	88,934
3.	("Takayasu Arteritis"[Majr] OR “Takayasu’s arteritis” OR “Granulomatous Vasculitis” OR “Pulseless disease”) AND “Tuberculosis"[Majr] OR Tuberculosis OR “Mycobacteria Tuberculosis” OR “TB” OR “active tuberculosis” OR “latent tuberculosis” OR “tubercul*."	26

**Table 2:** Search strategy in Cochrane Library and MedlinePlus

S. No.	Databases	Keywords	Search Results
1.	Cochrane Library	Takayasu’s arteritis and tuberculosis	4
2.	MedlinePlus	Takayasu’s arteritis and tuberculosis	3

Study Selection And Eligibility Criteria

After the completion of the search, we checked for duplicates. The relevant articles were screened through the titles and the abstracts by the two individual reviewers (MTM and SK). The articles published in the English language until April 2021 were only included. The articles documenting Takayasu’s arteritis associated with active or latent tuberculosis were included. Articles were excluded in cases of infection with non-tuberculous mycobacterial infection, unavailability of free full-text articles, overlapped with other articles, studies done on animals, and ones with incomplete data.

Results

**Figure 1:**
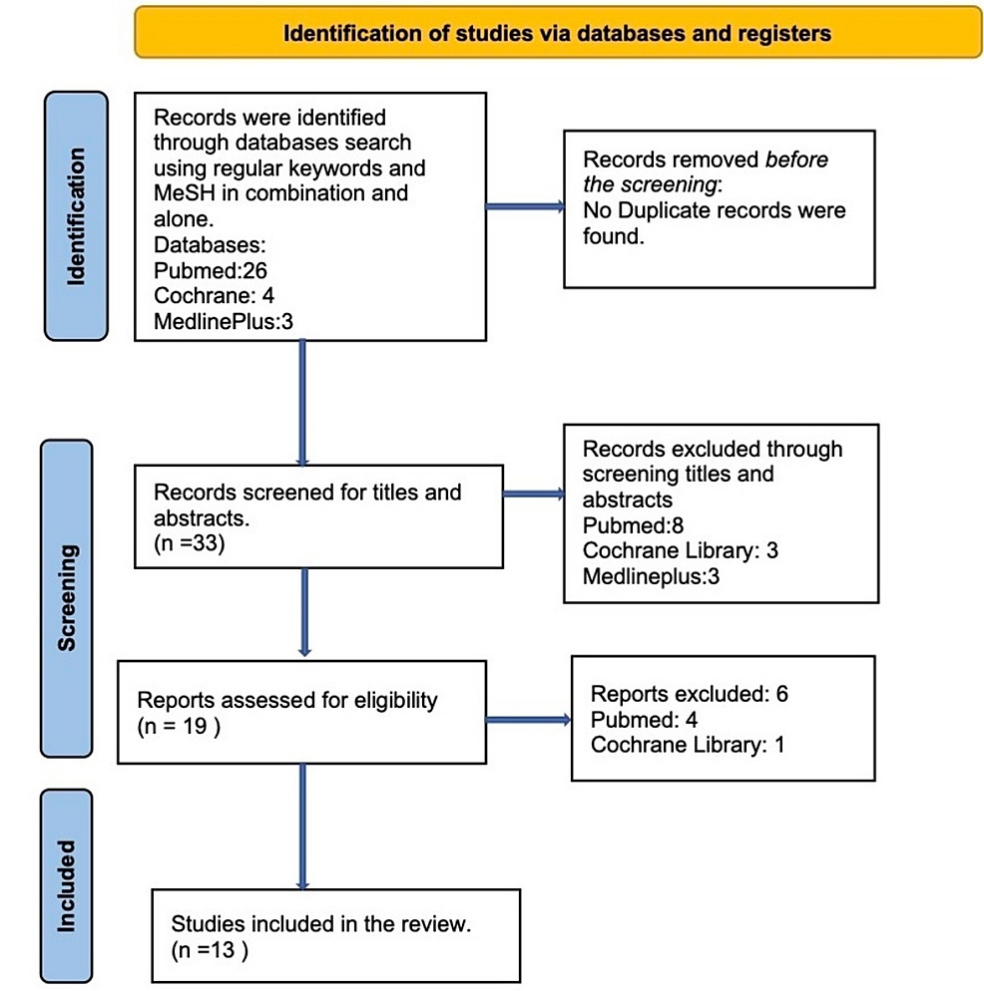
Flowchart of literature review search per Preferred Reporting Items for Systematic Reviews and Meta-analysis (PRISMA 2020) guidelines.

A total of 33 relevant articles were found, 26 from PubMed using the advance search strategy with a combination of regular keywords and MeSH terms, four from Cochrane Library, and three from MedlinePlus using the regular keywords. "Takayasu's arteritis" and tuberculosis. As there were no duplicates, the titles and abstracts of 33 articles were screened. Among the reviewed articles, only 19 articles were relevant to this research topic. Finally, these 19 articles were selected for review. Out of which only 13 were included for our study, six were excluded (three of them failed to meet the inclusion criteria, one was in a language other than English, and two were inaccessible). We assessed 13 studies for quality appraisal using standardized quality assessment tools, and all articles were qualified after the quality appraisal. The PRISMA flowchart of the literature and search strategy of the studies is shown in Figure 1 [18].

Study Characteristics

Table 3 shows the data extracted from the studies. Out of the 13 articles, eight were case reports, three were case-control studies, one cross-sectional, and one case series. The quality assessment tools that were used are mentioned as Joanna Briggs Institute (JBI) Checklist (case reports/case series) and Newcastle-Ottawa Checklist (observational studies).

**Table 3:** Data extraction table Y: years; F: female; M: male; TAK: Takayasu's arteritis; TB: tuberculosis; HBV: hepatitis B virus; PPD: purified protein derivative; M.Tb: *Mycobacterium tuberculosis*

S. No.	Author	Year	Study Design	TAK Cases	Age/Sex	Conclusion
1.	Mangouka et al. [17]	2020	Case report	1	Adult (39Y/F)	First case in Gabon who developed TAK associated with latent TB
2.	Agostinis et al. [19]	2019	Case report	1	Adult (75Y/M)	A TAK case with latent TB showed a significant reduction in thickened arteries when treated with only isoniazid 300mg for two weeks, which suggested isoniazid-induced TAK remission.
3.	Liebscher et al. [20]	2017	Case report	1	Adult (56Y/F)	This case report added to the concept of a potential association of TB infection and HBV infection with the pathogenesis of TAK, as treatment of these infections appeared to improve TAK symptoms.
4.	Carvalho et al. [21]	2017	Case-control study	52	Adults (mean age 35.2Y)	This study could not find mycobacterial DNA in peripheral blood mononuclear cells or tissue samples from the arteries of patients with established TA, indicating that mycobacterial infection may trigger disease development. But, sustained infection is unnecessary to cause active inflammation in TAK.
5.	Zhang et al. [22]	2017	Case report	1	Adolescent (18Y/F)	Development of pulmonary TB 6 months after the definite diagnosis of TAK, suggesting that *M. tuberculosis* may contribute to TAK via its production of superantigens and the elevated proinflammatory cytokines.
6.	Clemente et al. [23]	2016	Retrospective study	71	36 (children younger than 10Y), 35 (teenagers aged 10-19Y), according to WHO definition.	High frequency of tuberculin skin test positivity found in these cases showing an association between Mycobacterium Tb and TAK. Heat shock protein 65-kDa found in mycobacteria might cross-react with the homologous protein present in the vascular wall of the host, triggering an immune response.
7.	Khemiri et al. [24]	2016	Case report	1	12Y/F	Clinical findings suggesting co-occurrence of active pulmonary Tb and type 1 TA in Tunisia.
8.	Moura et al. [25]	2015	Case report	1	23Y/F	Mycobacterium can trigger TAK development or co-infection, as both conditions have a high prevalence in the Brazilian population.
9.	Nooshin et al. [26]	2013	Cross-sectional study	15	11 F and 4 M	40.0% of patients had a positive PPD test which suggests an association between TAK and prior *Mycobacterium tuberculosis* exposure.
10.	Soto et al. [16]	2012	Case-control studies on autopsies	33	N/A	Higher frequency of IS6110 and HupB genes in TAK patients' aortic tissues suggests that arterial damage could be due to prior infection with *Mycobacterium tuberculosis*.
11.	Zaki et al. [27]	2011	Case report	1	9Y/M	A case of TAK with associated abdominal tuberculosis.
12.	Al-Aghbari et al. [11]	2010	Case report	1	18Y/F	A first documented case of TAK associated with TB in Yemen.
13.	Muranjan et al. [28]	2000	Case series	17	(5-10Y/M)	Mantoux or Bacille Calmette-Guerin (BCG) test was positive in 6 patients diagnosed with TAK. However, no active tuberculous lesions were present.

Quality Assessment

We performed a thorough quality assessment for the 13 confirmed articles using two standard tools: the JBI Checklist (n=9) and the Newcastle-Ottawa Checklist (n=4). 

JBI Checklist scores of seven and above out of eight and Newcastle-Ottawa Checklist scores of seven and above out of eight were considered high-quality articles. JBI Checklist scores and Newcastle-Ottawa Checklist scores between four to six out of eight were considered intermediate quality articles. And a score below four was deemed to be low quality for both the JBI Checklist and the Newcastle-Ottawa Checklist.

Out of eight case reports, two scored eight of eight, and six scored seven of eight on the JBI Checklist. One case series scored eight of eight on the JBI Checklist. All three observational studies scored seven of eight on the Newcastle-Ottawa Checklist. All the articles that satisfied high-quality scores on the JBI Checklist and Newcastle-Ottawa Checklist were included in the review.

Discussion

To better understand the role of *Mycobacterium tuberculosis* in patients diagnosed with Takayasu’s arteritis, we studied 13 previously published articles, which in total included 196 patients diagnosed with Takayasu’s arteritis [11,16,17,19-28]. Out of which 68 had either a prior or current tuberculous infection [11,16,17,19-28].

Latent Tuberculosis in TAK Patients

Over many years, many studies show shreds of evidence implicating the contribution of *Mycobacterium tuberculosis* in the pathogenesis of TAK [21]. Our studies found 65 cases of latent tuberculosis in patients diagnosed with TAK [11,16,17,19-23,26,28]. We discovered that the majority of these observational studies detected latent tuberculosis in a patient diagnosed with TAK. These studies used various methodologies such as the Mantoux tuberculin skin test, Interferon-g release assay (IGRA), and QuantiFERON-TB tests to detect latent tuberculosis in the study subjects [11,16,17,19-23,26,28].

A recent case report by Mangouka et al. in 2020 reported a case of TAK with latent tuberculosis, which was the first-ever case to be reported in Gabon [17]. Furthermore, Agostinis et al. and Liebscher et al. published a case report of TAK with latent tuberculosis in 2019 and 2017, respectively [19,20]. A 75-year-old male diagnosed with TAK and tuberculosis had bilaterally thickened carotid arteries on ultrasound examination, among other symptoms, which showed significant reduction after two weeks of only isoniazid therapy [19]. A case report by Liebscher et al. presented TAK and infection with *Mycobacterium tuberculosis*, and hepatitis B [20]. Therapy for these infections and methotrexate led to improvement in TAK symptoms [20]. The positive response to TAK symptoms after the treatment suggests the possible role of *Mycobacterium tuberculosis* in TAK development [20]. 

In 2017, Zhang et al. reported an unusual case of pulmonary tuberculosis diagnosed six months after TAK was diagnosed [22]. Clemente et al. conducted a retrospective observational study to describe TAK's clinical and angiographic features in 71 Brazilian children and adolescents [23]. Their research revealed a higher frequency of tuberculin skin test positivity in their patients than healthy Brazilian children, as reported by the Brazilian Institute of Geography and Statistics [23]. This finding hints at the prevalence of latent tuberculosis in a patient with TAK. Although the exact etiology could not be identified, Clemente et al. highlighted that the immune response in TAK could be a result of cross-reaction between homologous protein present in the vascular wall of the host and the mycobacterial heat shock 65-kD [23].

Similarly, a cross-sectional study conducted by Nooshin et al. found the level of purified-protein derivative >10mm in six out of 15 study subjects, stressing the association of latent TB in a patient with TAK [26]. Furthermore, in 2010, Al-Aghbari et al. demonstrated a particular case of TAK who had a strongly positive Mantoux test for TB [11]. This was the first-ever case of TAK associated with TB in Yemen [11]. Lastly, the findings of Muranjan et al. highlighted the correlation between infection and TAK pathogenesis, who detected positive tuberculin skin test or Bacille Calmette-Guerin (BCG) in six (35.2% ) out of 17 patients with TAK [28].

Based on this data from these observational studies, the co-occurrence between TAK and latent tuberculosis can be seen. Nevertheless, careful interpretation is required as positive purified protein derivative (PPD), and IGRA tests could be influenced by the immunosuppressive agents and corticosteroids regularly used in TAK treatment. Additionally, the false-positive reaction to PPD could be due to prior vaccination with BCG, which was not distinctly illustrated in many studies. 

Active Tuberculosis in TAK Patients

The articles on concomitant TAK and TB have been published very scarcely [24]. Our review could identify only three such co-occurrence. In 2016, Khemiri et al. reported a case of TAK in a 12-year-old girl of Tunisia who had simultaneous active pulmonary tuberculosis [24]. Her diagnosis of active pulmonary TB was made based on clinical symptoms such as fever and chronic cough [24]. And tests such as positive QuantiFERON-TB and Mantoux test (20mm) and radiological imaging showing multiple parenchymal nodes in both lungs with enlarged hilar lymph nodes [24]. In 2011, Zaki et al. reported a case of a nine-year-old boy who had TAK associated with abdominal tuberculosis [27]. He was treated for abdominal tuberculosis with anti-tuberculosis treatment as he had a strongly positive Mantoux test, enlarged abdominal lymph nodes, and elevated erythrocyte sedimentation rate (ESR) [27]. Another study by Moura et al. reported a case where the clinical and radiological features present were shared by TAK and tuberculous arteritis [25]. Although the distinct diagnosis of TAK or tuberculous arteritis could not be illustrated by their study, the patient significantly improved after treatment with rifampicin, isoniazid, pyrazinamide, and ethambutol for six months along with prednisolone [25]. Their research study could not distinguish whether TB could undeniably trigger autoimmune processes in TAK or the simultaneous occurrence of TAK, and TB could just be a mere coincidence as both diseases have a high prevalence in the Brazilian population [25].

A physician should acknowledge the relation of concurrent active tuberculosis occurrence in TAK. Early detection of the concomitant infection with TAK could aid a physician in managing such cases more effectively and lessen the severity of the disease and improve the patient’s quality of life.

Role of Anti-Tubercular Drug on TAK Prognosis

In the study of Khemiri et al., anti-tubercular drugs did not affect TA vasculitis and did not aid in the prevention of new relapses [24]. On the contrary, a case reported by Agostinis et al. showed complete remission of TAK symptoms after treatment with only isoniazid for two weeks [19]. Additionally, the case report by Moura et al. showed substantial improvement in symptoms after therapy with anti-tubercular drugs and steroids [25]. Correspondingly, Liebscher et al. reported a case of TAK with TB, which revealed positive results after treatment with anti-tubercular medications [20].

The effect of anti-tubercular drugs on TAK symptoms remains debatable among authors [24]. With an anti-tubercular regimen, some authors portrayed the case of a thorough reduction in TAK symptoms along with the complete return of the affected pulses [24]. Though, it is essential to note that most studies have combined corticosteroid and anti-tubercular drugs to treat co-occurrence of TA and active TB.

Mycobacterial Genes in TAK Patients

Soto et al. evaluated the prevalence of *Mycobacterium tuberculosis* genes in the autopsy of 70% of aortic tissue samples from TA patients, compared to 32% in patients with atherosclerosis and 82% in patients with tuberculosis [16]. The authors identified a significantly high prevalence of insertion sequence (IS)6110 and HupB genes in aortic biopsies of patients with TAK, supporting the probability that TAK could result from latent TB infection [16]. The authors hypothesized that arteritis could result from a direct TB infection in the vessel wall based on those findings [16]. Despite all the evidence of the association, one observational study has failed to obtain the relationship of *Mycobacterium tuberculosis* in the pathogenesis of TAK [21]. In 2016, a case-control study was conducted by Carvalho et al. to show the presence of three different mycobacterial nucleic acid sequences [21]. Namely, insertion sequence (IS) 6110, the 65-kDa heat shock protein gene (HSP65), and the 16S ribosomal RNA (rRNA) in peripheral blood from 32 TAK patients and arteries from 10 TAK patients [21]. However, their study failed to detect mycobacterial DNA in peripheral blood and arterial tissues of diagnosed TAK patients, thus lacking support for the association between TAK and TB [21]. They also concluded their study, indicating that mycobacterial infection is not required to maintain arterial inflammation in TAK, although it may trigger the initial development process of TAK [21].

In summary, we analyzed that many studies in our review showed a collection of indirect findings signifying a potential link between tuberculosis and TAK; however, only one failed to detect the aforementioned link. In these observational studies, most have latent tuberculosis in patients with TAK, suggesting that infection with *Mycobacterium tuberculosis* could trigger TAK development. Although the etiopathogenesis of TAK remains unclear, tuberculosis was hypothesized to be one of the prompting influences [28]. Karadag et al. suggested that the previously reported studies that addressed the connection between the prior exposure to *Mycobacterium tuberculosis* and TAK patients may be considered incidental in countries where TB is endemic [29].

The direct role of *Mycobacterium tuberculosis* is not entirely suggested as current literature hypothesizes autoimmunity involving both cellular and humoral factors as a chief contributor to TAK 's pathogenesis [24,28]. Still, various other factors such as genetic predisposition, post-infective, and ethnic susceptibility have also been considered [24,28].

Limitation

The present systematic review certainly has some limitations. The major one is the selection of studies was conducted in three databases with a limit of language (English), so some studies with the same subject could likely be left out. We included only free full-text articles, so many informative studies that appeared relevant during screening might have been missed resulting in obliteration of our overall review. Furthermore, most of our included studies have a small population of participants and a small number of prior published articles that could affect our research.

## Conclusions

This systematic review aimed to emphasize the linkage of *Mycobacterium tuberculosis* in the etiopathogenesis of Takayasu's arteritis. Furthermore, the goal was to study the possible cause of this link. As a casual connection has not been established yet, it is difficult to say that either latent or active tuberculous infection can lead to TAK. However, as most of the studies in our review favor this association, both in the adult and pediatric population, it can be hypothesized that either latent or active *Mycobacterium tuberculosis* infection has to be among one trigger for TAK. Nonetheless, more studies are required to explore the exact role of *Mycobacterium tuberculosis* in the etiopathogenesis of Takayasu's arteritis.
